# Supplementary material for: Overcoming host immune responses to an AAV-delivered HIV-1 bNAb in rhesus macaques mediated by co-delivery of PD-L1
Source: bioRxiv. 2026 Jun 1:2026.05.29.728806. Preprint. [Version 1] doi: 10.64898/2026.05.29.728806 (PMC13251908; doi:10.64898/2026.05.29.728806)
Supplement: Supplement 1 [file NIHPP2026.05.29.728806v1-supplement-1.pdf]

bioRxiv preprint doi: https://doi.org/10.64898/2026.05.29.728806; this version posted June 1, 2026. The copyright holder for this preprint (which was not certified by peer review) is the author/funder, who has granted bioRxiv a license to display the preprint in perpetuity. It is made available under a CC-BY 4.0 International license.

Figure S1. Macaque weight gain over the course of the study.

Weight gain over the course of 52 weeks in macaques that received **(A)** AAV9.10-1074 only or **(B)** AAV9.10-1074 plus AAV9.PD-L1.

Table S1: Characteristics of the 12 rhesus macaques enrolled in this study.

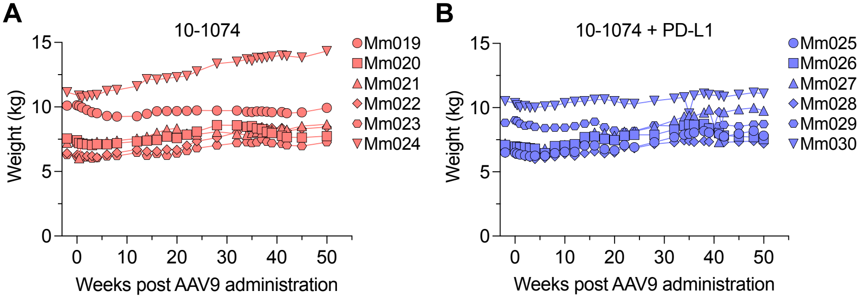

Kuipa et al., Fig. S1

Macaque code	Group	Age (years)	Weight (kg)	Sex	*Mamu-B*17*	*Mamu-B*08*	*Mamu-A*01*	*Mamu-A*02*
Mm019	10-1074-only	9.7	10.11	F	-	-	+	-
Mm020	10-1074-only	3.8	7.55	M	-	-	+	-
Mm021	10-1074-only	3.8	7.23	M	-	-	-	-
Mm022	10-1074-only	4.0	6.42	M	-	-	-	-
Mm023	10-1074-only	3.8	6.28	M	-	-	-	-
Mm024	10-1074-only	4.9	11.14	M				
Mm025	10-1074 plus PD-L1	3.8	6.5	M	-	-	-	-
Mm026	10-1074 plus PD-L1	4.8	7.1	M	-	-	-	-
Mm027	10-1074 plus PD-L1	3.8	7.22	M	-	-	-	-
Mm028	10-1074 plus PD-L1	3.8	6.48	M	-	-	-	+
Mm029	10-1074 plus PD-L1	6.0	8.82	F	-	-	-	-
Mm030	10-1074 plus PD-L1	6.7	10.5	F	-	-	-	-

Kuipa et al., Table S1
